# Cost-effectiveness of strategies to improve the utilization and provision of maternal and newborn health care in low-income and lower-middle-income countries: a systematic review

**DOI:** 10.1186/1471-2393-14-243

**Published:** 2014-07-22

**Authors:** Lindsay Mangham-Jefferies, Catherine Pitt, Simon Cousens, Anne Mills, Joanna Schellenberg

**Affiliations:** 1 Department of Global Health and Development, London School of Hygiene and Tropical Medicine, London, UK; 2 Department of Infectious Disease Epidemiology, London School of Hygiene and Tropical Medicine, London, UK; 3 Department of Disease Control, London School of Hygiene and Tropical Medicine, London, UK

**Keywords:** Cost-effectiveness, Strategy, Intervention, Behaviour change, Service delivery, Maternal and newborn health care, Low-income countries, Lower-middle-income countries

## Abstract

**Background:**

Each year almost 3 million newborns die within the first 28 days of life, 2.6 million babies are stillborn, and 287,000 women die from complications of pregnancy and childbirth worldwide. Effective and cost-effective interventions and behaviours for mothers and newborns exist, but their coverage remains inadequate in low- and middle-income countries, where the vast majority of deaths occur. Cost-effective strategies are needed to increase the coverage of life-saving maternal and newborn interventions and behaviours in resource-constrained settings.

**Methods:**

A systematic review was undertaken on the cost-effectiveness of strategies to improve the demand and supply of maternal and newborn health care in low-income and lower-middle-income countries. Peer-reviewed and grey literature published since 1990 was searched using bibliographic databases, websites of selected organizations, and reference lists of relevant studies and reviews. Publications were eligible for inclusion if they report on a behavioural or health systems strategy that sought to improve the utilization or provision of care during pregnancy, childbirth or the neonatal period; report on its cost-effectiveness; and were set in one or more low-income or lower-middle-income countries. The quality of the publications was assessed using the Consolidated Health Economic Evaluation Reporting Standards statement. Incremental cost per life-year saved and per disability-adjusted life-year averted were compared to gross domestic product per capita.

**Results:**

Forty-eight publications were identified, which reported on 43 separate studies. Sixteen were judged to be of high quality. Common themes were identified and the strategies were presented in relation to the continuum of care and the level of the health system. There was reasonably strong evidence for the cost-effectiveness of the use of women’s groups, home-based newborn care using community health workers and traditional birth attendants, adding services to routine antenatal care, a facility-based quality improvement initiative to enhance compliance with care standards, and the promotion of breastfeeding in maternity hospitals. Other strategies reported cost-effectiveness measures that had limited comparability.

**Conclusion:**

Demand and supply-side strategies to improve maternal and newborn health care can be cost-effective, though the evidence is limited by the paucity of high quality studies and the use of disparate cost-effectiveness measures.

**Trial registration:**

PROSPERO_ CRD42012003255.

## Background

Worldwide, each year 3 million newborns die within the first 28 days of life [1], 2.6 million babies are stillborn [2], and 287,000 women die from complications of pregnancy and childbirth [3]. The vast majority of these deaths occur in Africa and Asia, many could be prevented by improving access to existing interventions [1-3]. Substantial evidence exists on a wide range of interventions and behaviours, and reviews have identified life-saving maternal and newborn health (MNH) interventions that are not only effective, but also cost-effective and suitable for implementation in resource-constrained settings [4-6]. Examples include iron supplements to prevent anaemia, tetanus toxoid immunization, magnesium sulphate for eclampsia, uterotronics to prevent and manage post-partum haemorrhage, hygienic cord care, immediate thermal care, exclusive breastfeeding, and management of neonatal sepsis, meningitis and pneumonia [6]. It has been estimated that increased coverage and quality of pre-conception, antenatal, intra-partum, and post-natal interventions by 2025 could avert 71% of newborn deaths [5].

Despite efforts to identify priority interventions, access to life-saving MNH interventions remains inadequate in low and middle income countries [6]. There may be delays making the decision to seek care, difficulties in reaching care, or problems with the range or quality of care available [7]. To achieve higher coverage of MNH interventions, effective and cost-effective strategies need to be identified that address these challenges and lead to improvements in the utilization and provision of MNH care. Demand-side strategies are needed to influence health practices of individuals and communities and promote uptake of preventive and curative MNH care during pregnancy, childbirth and in the post-natal period. These strategies may provide health information and education, or address geographic, financial, or cultural barriers to accessing care. At the same time, supply-side strategies are needed to enhance the capability and performance of front-line health workers, who as the first point-of-contact for women and newborns provide essential care in the community and at primary health facilities. Supply-side strategies may involve training to ensure health workers have the necessary knowledge and skills, and strategies to motivate health workers, improve the working environment and resources available, or strengthen other aspects of the health system.

Several recent reviews have summarized the evidence on the effectiveness of these strategies to improve MNH care in low and middle income countries [8-14]. The reviews range in scope and setting, and have focused on community-based strategies, integrated primary care, and urban settings. However, given resource constraints, it is important to know not only what strategies are effective at improving coverage of MNH interventions, but also whether the strategies are cost-effective. This has been highlighted as a research priority [12], and the existing reviews contain only limited information on cost-effectiveness [9,11,12,14].

This paper presents a systematic review of the cost-effectiveness of strategies to improve the demand and supply of maternal and newborn health care in low-income countries (LICs) and lower-middle-income countries (LMICs). Our focus is not the interventions and behaviours themselves, but strategies for ensuring they are made available and taken up. The protocol for the systematic review was registered with the International Prospective Register of Systematic Reviews (PROSPERO) [15].

## Methods

### Searches

**Figure 1:**
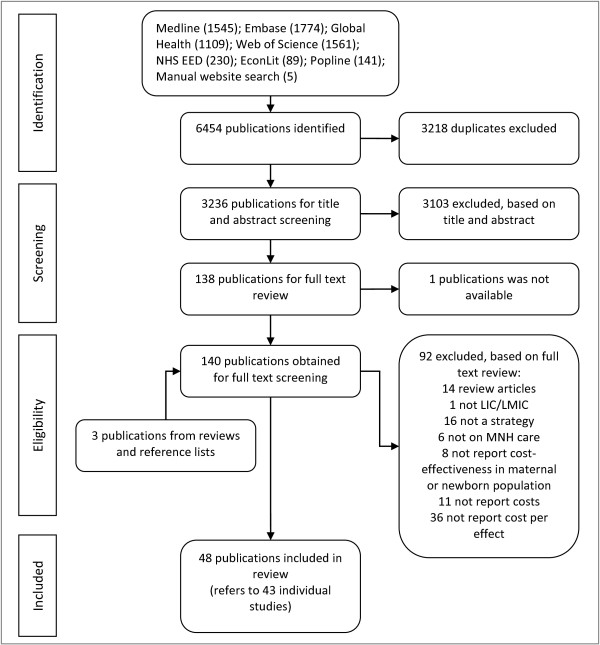
Flow diagram for selection of studies.

The title and abstract of peer-reviewed and grey literature published since 1 January 1990 were searched using terms (including synonyms and MeSH terms) for three concepts: i) cost-effectiveness, ii) MNH care, and iii) LICs and LMICs (Additional files 1 and 2). Searches were conducted in six electronic bibliographic databases, Medline, Embase, Global Health, EconLit, Web of Science, and the NHS Economic Evaluation Database (Additional file 1) on 14 September 2012 and last updated on 16 October 2013. Grey literature was searched using the Popline database and websites of selected organizations and networks, including the Partnership for Maternal, Newborn and Child Health, Maternal Health Task Force, Healthy Newborn Network, UNICEF, and World Health Organization (Additional file 1). This initial search identified 3236 non-duplicate publications that were eligible for title and abstract screening (Figure 1). The reference lists of reviews identified in the title and abstract search and included publications were also screened.

### Article selection and exclusion criteria

The process of study selection is summarized in Figure 1. The title and abstract of the retrieved citations were uploaded into EPPI Reviewer 4 software [16] and independently screened by two reviewers (LMJ and CP). Publications that did not clearly meet criteria for exclusion were retained for full text review. The full text of retained publications was then independently assessed for exclusion criteria by both reviewers and any reasons for exclusion recorded. Discrepancies between reviewers were relatively few and were resolved through discussion without the need to consult a third reviewer.

At the title and abstract review stage, publications were excluded if they fulfilled any of the following criteria:

• Did not report on a strategy that sought to influence health practices or enhance front-line worker performance.

• Did not report on maternal or newborn care.

• Was not set in a LIC or LMIC.

• Did not report on costs.

• Did not report the effect of the strategy.

• Was published before 1990.

• Was a letter or editorial.

At the full-text review stage, publications were excluded if they met any of the previous criteria, or, additionally, if they:

• Did not report a cost-effectiveness measure.

• Presented secondary rather than primary analysis (in which case the references were checked for additional articles for inclusion).

The definitions used for “cost-effectiveness measure”, “strategies”, “maternal and newborn health care”, and “LICs and LMICs” are provided in Table 1.

**Table 1:** Key definitions used in search strategy

The systematic review aimed to identify studies that report on the **cost-effectiveness** of **strategies** to improve the utilization and/or provision of **maternal and newborn health care** in **low-income and lower-middle-income countries**. We define the key terms here:	
•	**Cost-effectiveness measure:**
	reports a measure of cost per effect. Articles were eligible if they reported on the cost per health care interaction, per biomedical intervention received, per health gain, per life-saved (or death averted), per life-year saved (or years of life lost averted), per quality adjusted life-year (QALY) gained, or per disability-adjusted life-year (DALY) averted. The cost per effect measure was defined broadly, though it did need to refer to the population benefiting from maternal and newborn care. For example, strategies to deliver mosquito nets were included only if the cost-effectiveness measure reported on the effect on pregnant women or their newborns.
•	**Strategies:**
	one or more innovations, initiatives, approaches, or activities that aim to either i) influence health practices of individuals or communities or ii) improve health care provision by enhancing front-line worker capability and performance. Strategies may target the individual, community, front-line worker, organization or another aspect of the health system. Articles on biomedical interventions (e.g. prevention of mother-to-child transmission of HIV or administering misoprotosol) were eligible only if they contained one more activities that sought to directly change its demand or supply. For instance, an article on a new syphilis test would be eligible only if there were also activities to support its introduction, such as training frontline workers in how to prevent and manage syphilis in pregnant women.
•	**Maternal and newborn health (MNH) care:**
	an interaction that takes place between a front-line worker (of any cadre) and a woman, newborn or other family member during pregnancy, childbirth or in the 28 days following birth.
•	**Low-income countries (LICs) and lower-middle-income countries (LMICs):**
	countries with a gross domestic product (GDP) per capita of less than US $4036, as categorised by the World Bank in 2012. LICs have GDP per capita less than US $1026, and LMICs have a GDP per capita between US $1026 and US $4035. A list of eligible countries is provided in Additional file 2.

### Data extraction

A standardized form was used to extract the data required for the quality assessment and evidence synthesis. The first reviewer (LMJ) completed the data extraction form for all studies, and the second reviewer (CP) assessed the accuracy of the extracted data and quality assessment. Differences were resolved through discussion.

### Quality assessment

Study quality was assessed using the Consolidated Health Economic Evaluation Reporting Standards (CHEERS) statement [17], which was published in 2013. The CHEERS statement contains a checklist of 24 criteria intended to establish the minimum information that should be included when reporting economic evaluations of health strategies and interventions and each publication included in the review was assessed against these criteria.

To obtain an overall quality assessment, publications scored 1 point for each criterion fully met, 0.5 for each partially met and 0 for each when no or very little information was reported. A percentage score was then generated, giving all criteria equal weight (criteria not applicable were excluded from the calculation). Studies that scored 75% or more were categorized as high quality, scores in the range 50-74% were ranked medium, and scores less than 50% were ranked poor. As two of the reporting criteria may depend on the publisher (source of funding and conflicts of interest), we also calculated the percentage score excluding these criteria and found this had no effect on the categorization.

### Data synthesis

Descriptive information about the eligible studies was summarized using text and tables. The demand- and supply-side strategies were listed alongside a brief description, its comparator, and whether it focused on a specific aspect of MNH care (Table 2). The study design, study year and primary outcome measure are also listed, and the effect of the strategy is included when a relative risk, odds ratio or proportion by study arm was reported.

**Table 2:** Overview of included studies

**Country**	**Strategy**	**Description of strategy**	**Type of MNH care**	**Study design**	**Study year**	**Magnitude/Measure of effect**	**Author (Year)**	**Reference**
Bangladesh	Women’s groups & health system strengthening (HSS)	Recruit & train facilitators to convene monthly women’s group meetings. Women’s groups encouraged to adopt strategies to improve MNH. In all clusters basic medical equipment supplied, TBAs trained in essential newborn care, physicians trained, and links established between communities and health services.	MNH, especially newborn	Cluster RCT	2009- 2011	NMR per 1000 LB Adj RR = 0.62 (95% CI: 0.43-0.89)	Fottrell (2013)	[18]
Bangladesh	Vouchers for free MNH care, cash and in-kind transfers	Family welfare assistants (FWAs) distribute vouchers to (eligible) pregnant women entitling them to free ANC, delivery, emergency referral & post-partum care. Cash stipends for transport & other costs. Cash incentive to deliver in facility or at home with skilled provider. In-kind items (including soap and newborn clothes) supplied. Providers reimbursed for services provided.	All MNH	Intervention & control areas	2008- 2009	% of deliveries with qualified provider: 58-70% (S), 27% (C)	Hatt (2010)	[19]
Bangladesh	Improve health and family welfare clinics	Staff & equip primary health facilities to provide delivery & newborn care (incl. additional staff, improved infrastructure, equipment and supplies).	Facility-birth	Cost projection	2011	Estimated number of clients per year	Howlander (2011)	[20]
Bangladesh	Promotion of NGO health clinics:	Smiling Sun communication campaign to promote NGO clinics (incl. 26 episode TV drama, TV adverts, radio spots, posters, billboards, adverts in daily newspapers, & local publicity efforts).	Facility-based ANC	Secondary data analysis	2001- 2004	Estimated number of new ANC users	Hutchinson (2006)	[21]
	1. National media campaign							
	2. National media campaign & local activities							
Bangladesh	MNH services delivered at home, with community mobilization and health system strengthening (HSS)	CHWs recruited and trained to conduct home visits during pregnancy and post-natal period (incl. treatment of newborns with antibiotics). Communities were mobilized. HSS in all clusters: facility-level providers trained in MNH care, drugs & supplies distributed, & system for tracking neonatal care established.	All MNH	Cluster RCT	2003- 2005	NMR per 1000 LB: 31.2 (S), 43.1 (C)	LeFevre (2013)	[22]
Bangladesh	Outreach clinics for FP and ANC by facility staff†	Increase number of outreach clinics and extend opening hours. Compared to services (ANC care) provided at primary care facilities.	Pregnancy	Intervention & control areas	1996- 1997	Number of ANC services provided	Levin (1997 & 1999)	[23,24]
Bangladesh	Alternative delivery strategies FP & MCH	1. FP & MCH services by government fieldworkers at community service points (e.g. clubs, schools);	FP & MCH	Intervention & control areas	1996- 1997	Number of ANC services provided	Routh (2000)	[25]
		2. FP & MCH services provided at primary care facility Compared to FP & MCH services provided at home by government fieldworkers.						
Bangladesh	Train TBAs	TBAs trained and encouraged to refer difficult births.	Intra-partum	Estimated from secondary data	Not given	Neonatal lives saved per 1000 LB: 7 (estimate)	World Bank (2005)	[26]
Benin & Guinea	Bamako Initiative	Various strategies to improve primary care, incl. integrated minimum package of preventive and curative MNCH care.	ANC (incl. prophylaxis for malaria & anaemia)	Secondary data analysis	1989- 1993	% of pregnant women having at least 3 ANC visits: 43% Benin, 55% Guinea	Soucat (1997)	[27]
Burkina Faso	Train new cadres in EmOC	Develop and implement training:	EmOC	Pre-Post	2004- 2007	Newborn case fatality rate per 1000 c-sections: 125 (S1), 198 (S2), 99 (C)	Hounton (2009)	[28]
		S1. Six-months in essential surgery for medical doctors,						
		S2. Two-years in surgery (incl. C-section) for clinical officers.						
		C. Compared to training obstetricians						
Burkina Faso	Initiative to promote facility-birth	Recruit & train of community link workers. Community mobilization by traditional leaders to promote assisted delivery. Also PHC staff trained, equipment supplied & infrastructure improved.	Facility-birth	Intervention & control areas	2002- 2005	20.8% point increase in births delivered in a facility	Newlands (2008) & Hounton (2012)	[29,30]
Cambodia	Introduce HIV testing:	Facilities supplied HIV test kits. Five-day HIV training for midwives. Facility paid per person tested, and per person counselled and referred. Monthly staff meetings and regular supervision.	HIV testing & mgmt	Pre-Post	2008- 2009	% tested for HIV: 97.6% at ANC, 95% at labour	Heller (2011)	[31]
	1. at ANC							
	2. at labour							
Cambodia	Community health education by midwives	Midwives trained to lead focus group discussions to engage community on maternal health.	Birth preparedness	Pre-Post	2005- 2006	22% increase in ANC, 32% increase births with midwife, 19% decrease in women using TBAs, 281% increase in referrals	Skinner (2009)	[32]
Democratic Republic of Congo	Distribute malaria ITNs at ANC	Nurses trained to distribute ITNs to pregnant women attending ANC.	Malaria prevention	Economic model (incl. primary data)	2005- 2006	Number of ITNs delivered, and estimated number of infant deaths averted	Becker-Dreps (2009)	[33]
The Gambia	Outreach maternal health care	Outreach ANC clinics ran by midwives & community nurses. Activities to identify pregnant women, support for referral). Also train TBAs (incl. 6 month refresher). New fixed price for MNH care.	Pregnancy & Intra-partum	Intervention & control areas	1989- 1991	NMR per 1000 LB: 16 (S), 32.2 (C) MMR per 1000 LB: 3.1 (S), 7 (C)	Fox-Rushby (1995 & 1996)	[34,35]
Honduras	Hospital-based promotion of breastfeeding	Hospital staff trained to educate & encourage mothers to breastfeed. Included changes to establish early breastfeeding contact, rooming-in of babies with mothers, withdrawal of routine bottle feeding, & post-partum counselling.	Promote breastfeeding	Intervention & control areas	1992- 1993	% exclusive breastfeeding: 42.7% (S), 22.2% (C)	Horton (1996)	[36]
						Neonatal deaths averted per 1000: 1.02 (from acute respiratory infection), 3.48 (from diarrhoea)		
India	Women’s groups & health system strengthening (HSS)	Recruited & trained facilitators to convene monthly women’s group meetings. Women’s groups encouraged to adopt strategies to improve MNH. HSS (incl. training in newborn care, equipment & supplies) in all areas.	All MNH	Cluster RCT	2005- 2008	NMR per 1000 LB: 42.9 (S), 59.1 (C); Adj OR = 0.71 (95% CI: 0.61-0.83)	Tripathy (2010)	[37]
India	HIV testing at ANC:	Activities include: health education using local media campaign and HIV testing, counselling, drug treatment at ANC.	HIV testing & management	Economic model	2005	Estimated number of cases of perinatal HIV prevented	Kumar (2006)	[38]
	1. nationwide							
	2. in high prevalence states							
India	Home-based neonatal care by Village Health Workers (VHWs)	Recruit and train female VHWs to identify & counsel pregnant women, & to undertake home-based neonatal care. Also VHWs may support TBAs at delivery. VHWs were supervised every 2 weeks.	All MNH, esp. thermal care, birth asphyxia, breastfeeding, neonatal sepsis	Pre-Post + Control	1993- 2003	70% reduction in NMR per 1000 LB: 62 (Pre), 25 (Post)	Bang (2005a)	[39]
India	Home-based management of birth asphyxia by Village Health Workers (VHWs)	Trained female VHWs to diagnose and manage birth asphyxia (when support TBAs at delivery) Compared to current practice (with TBAs trained to manage birth asphyxia.	Birth asphyxia	Pre-Post	1996- 2003	65% reduction in NMR per 1000 LB: 10.5 (Pre), 3.6 (Post)	Bang (2005b)	[40]
India	Home-based neonatal care by Village Health Workers (VHWs)	Recruit and train female VHWs to identify & counsel pregnant women, & to undertake home-based neonatal care. Also VHWs may support TBAs at delivery. VHWs were supervised every 2 weeks.	All MNH, esp. thermal care, birth asphyxia, breastfeeding, neonatal sepsis	Pre-Post + Control	1993- 1998	62% reduction in NMR per 1000 LB in year 3: 25.5 (S), 59.6 (C)	Bang (1999)	[41]
Indonesia	Tetanus toxoid (TT) immunization campaign	TT immunization campaign by new nursing graduates, supported by community mobilization using village heads and women’s groups. Compared to TT immunization at routine ANC.	Tetanus toxoid immunization	Intervention & control areas	1985	Number of women who received full TT dose, and estimated number of neonatal (tetanus) deaths averted	Berman (1991)	[42]
Kenya	Distribute malaria ITN at ANC	Facilities instructed to procure ITNs and distribute to pregnant women during ANC.	Malaria prevention	Prospective	2001	Reports number ITNs distributed. 77% were to pregnant women	Guyatt (2002)	[43]
Kenya	Syphilis testing at ANC.	On-site syphilis testing using rapid syphilis test, on-site same day treatment of RPR-positive, promotion of notification and presumptive treatment of women’s partners.	Syphilis testing & treatment	Pre-Post	1998- 2000	% clients at ANC screened: 81% (S1), 51% (S2)	Population Council (2001)	[44]
	1. on-site							
	2. standard clinics (off-site)							
Kenya	Decentralized programme of syphilis control	Programme included: laboratory support; supplies and drugs; training ANC nurses in rapid syphilis testing and treatment of seroactive women; counselling; partner notification; supervision & monitoring.	Syphilis diagnosis and treatment	Pre-Post	1997- 1998	Number of women screened, number test-positive, & number treated	Fonck (2001)	[45]
Kenya	Decentralized programme of syphilis control	Programme included: laboratory support; supplies and drugs; training ANC nurses in rapid syphilis testing and treatment of seroactive women; counselling; partner notification; supervision & monitoring.	Syphilis testing & mgmt	Pre-Post	1992- 1993	Number of syphilis cases treated, and estimated number of congenital syphilis averted	Jenniskens (1995)	[46]
Malawi	1. Women’s groups	1. Recruited & trained facilitators to convene women's groups. Women's groups encouraged to identify and adopt local strategies to improve MNH.	All MNH	Factorial Cluster RCT	2008- 2010	NMR per 1000 LB: 27.0 (S3), 34.0 (C); Adj OR = 0.78 (95% CI: 0.60-1.01)	Colbourn (2013a & 2013b)	[47,48],
	2. Quality improvement at health facilities,							
	3. Both 1 & 2	2. Facility staff trained to initiate and run quality improvement initiative. Facility staff to identify & adopt local strategies to improve facility-based services (e.g. staff training needs).						
Malawi	1. Women’s groups	1. Recruited & trained facilitators to convene women's groups. Women’s groups encouraged to identify and adopt local strategies to improve MNH. 2. Volunteer peer counsellors made home visits during pregnancy and post-birth to support breastfeeding and infant care.	All MNH	Factorial Cluster RCT	2005- 2009	Factorial analysis: NMR per 1000 LB (S1 vs C): OR = 0.85 (95% CI: 0.59-1.22) MMR per 100,000 LB (S1 vs C): OR = 0.48 (95% CI: 0.26-0.91) IMR per 1000 LB (S2 vs C): OR = 0.89 (95% CI: 0.72-1.10)	Lewycka (2013)	[49]
	2. Peer counselling							
	3. Both 1 & 2							
Mozambique	Train Assistant Medical Officers in Emergency Obstetric Care (EmOC)	Two-year classroom-based instruction followed by 1-year internship. (In comparison, physicians receive 6-years of medical training and 5-year residency in surgery and obstetrics).	EmOC	Economic model (primary data)	2004	Number of obstetric surgeries performed	Kruk (2007)	[50]
Nepal	Women’s groups & health system strengthening (HSS)	Recruited & trained facilitators to convene monthly women’s group meetings. Women’s groups encouraged to adopt strategies to improve MNH (e.g. community-fund, stretcher schemes, clean delivery kits, home visits). HSS (incl. training in newborn care, equipment & supplies) in all areas.	All MNH	Cluster RCT	1999- 2003	NMR per 1000 LB 26.2 (S), 36.9 (C).	Borghi (2005)	[51]
						Adj OR = 0.70 (95% CI: 0.53-0.94)		
Niger	Quality improvement collaborative	Used facility data to monitor indicators of common technical interventions. Staff worked collaboratively to identify strategies to overcome service delivery barriers and improve facility care.	Intra-partum/Post-partum care (incl. AMTSL & PPH)	Pre-Post	2006- 2008	MMR per 10,000 vaginal births (projected:) 7.11 (Pre), 0.98 (Post)	Broughton (2013)	[52]
Niger	Programme to treat obstetric urogenital fistula	Programme include hospital stay, hygiene education, medical and surgical treatment and social rehabilitation interventions. All provided free of charge.	Obstetric urogential fistula	Pre-Post	2006	Number of women benefitting from the programme during study period	Ndiaye (2009)	[53]
Nigeria	Establish and train community contact persons	Select and trained contact persons to provide community health education, visit pregnant women, & facilitate referral (if needed).	Pregnancy; & support referral	Pre-Post	1993- 1995	Number of women assisted by contact persons during study period	Nwakoby (1997)	[54]
Nigeria	Establish emergency transport scheme	Mobilize transport union, drivers given basic training & awareness on health topics. Also seed money to establish revolving petrol fund.	Emergencies (pregnancy & intra-partum)	Pre-Post	1994- 1995	Number of obstetric emergencies transported during study period.	Shehu (1997)	[55]
Papua New Guinea	Improve standard of special neonatal care	Special care nurses trained on management of neonatal illnesses, including new treatment protocol for low-birth weight babies. Special care units provided equipment (e.g. pulse oximetry). Also clinical supervision and a weekly mortality audit.	Special neonatal care (incl. mgmt birth asphyxia, neonatal sepsis, pneumonia)	Pre-Post	1995- 2000	In-hospital neonatal mortality: RR = 0.56 (95% CI: 0.45-0.69)	Duke (2000)	[56]
Senegal	Remove user fees for intra-partum care	Removed user fees for intra-partum care (including caesarean section) in poor regions.	Facility-birth (incl. c-section)	Pre-Post	2004- 2006	% births supervised by normal delivery: 40% (Pre), 44% (Post)	Witter (2010)	[57]
						% of births by C-section 4.2% (Pre), 5.6% (Post)		
Uganda	Compare four strategies for abortion care	Alternative strategies are defined along two dimensions: availability and type of practice:	Abortion care	Economic Model	1996	Not applicable estimates cost per abortion case	Johnston (2007)	[58]
		1 Restricted-conventional,						
		2. Restricted-recommended,						
		3. Liberal-conventional, 4. Liberal-recommended. Also examined strategies at different levels of care.						
Uganda	Home-based distribution of intermittent preventive treatment in pregnancy (IPTp)	Community resource persons trained to identify pregnant women, make home visits and distribute IPTp, folic acid and iron supplements. Compared to IPTp distributed at PHC during ANC.	Malaria prevention (IPTp)	Prospective	2003- 2005	% of women with anaemia: 49% (S), 41% (C) % of LBW babies: 8% (S), 6% (C)	Mbonye (2008a & (2008b)	[59,60]
Uganda	Establish emergency transport	Established local ambulance service available 24 hours.	Emergencies (pregnancy & intra-partum)	Pre-Post	2009- 2010	Number of obstetric referrals during study period	Somigliana (2011)	[61]
Ukraine	Initiative on evidence-based practice in maternal and infant hospital care	Eight-year project advocating reduction in (elective) c-sections and evidence-based medical practices (incl. amniotomies and episiotomies, early breastfeeding, skin-to-skin contact, rooming in). Maternity staff trained & sought to develop centres of excellence.	Intra-partum & newborn care	Pre-Post	2002- 2005	4.71% reduction in number of (elective) C-sections	Nizalova (2010)	[62]
Zambia	Syphilis testing at ANC	Facilities supplied syphilis tests. Five-day training on pregnancy care (incl. syphilis). Also used community health education via local leaders to improve ANC attendance.	Syphilis testing & mgmt	Pre-Post + Control	1986- 1987	% of adverse pregnancy outcomes among seroreactive women: 28.3% (S), 72.4% (C)	Hira (1990)	[63]
Zambia	Train midwives in newborn care	Clinic midwives trained using 5-day WHO course on neonatal care and management of neonatal illnesses.	Newborn care (incl thermal care & breastfeeding)	Pre-Post	2004- 2006	NMR per 1000 (by 7-day): 11.5 (Pre), 6.8 (Post) RR = 0.59 (95% CI: 0.48–0.77)	Manasyan (2011)	[64]
Zambia	Train TBAs & supply clean delivery kits	TBAs trained over 4-day training (with refresher training every 3–4 months), provided equipment and clean delivery kits.	Intra-partum & newborn care	Cluster RCT	2006- 2008	Estimated number of neonatal deaths averted	Sabin (2012)	[65]

^†^Note: The study also compared provision of family planning to groups of women at a centrally located house with home-based doorstep strategy.

Acronyms used: *Adj*: adjusted; *ANC*: antenatal care; C: comparator; *CHW*: community health worker; *CI*: confidence interval; *C*-section: caesarean section; *EmOC*: emergency obstetric care; excl.: excluding; *FP*: family planning; *FWA*: family welfare assistant; *HSS*: health system strengthening; *IMR*: infant mortality rate; *Incl*.: including; *ITNs*: insecticide-treated bed nets; *IPTp*: intermittent preventive treatment in pregnancy; *LB*: live births; *LBW*: low birth weight; *MCH*: maternal and child health; *MNCH*: maternal, newborn, and child health; *MNH*: maternal and newborn health; *MMR*: maternal mortality rate; *NGO*: non-governmental organisation; *NMR*: neonatal mortality rate; *OR*: odds ratio; *PHC*: primary health care; *RCT*: randomized control trial; RR: relative risk; S: strategy; S1: strategy 1; S2: strategy 2; *TBA*: traditional birth attendant; *TT*: tetanus toxoid; *VHWs*: village health workers; *WHO*: World Health Organization.

Narrative synthesis was used to analyse and interpret the findings. Across the studies common themes were identified, based on the authors’ description of the strategy, whether it was implemented in the community, primary care facilities or hospitals, and whether it was specific to pregnancy, intra-partum, post-partum or post-natal care or applied to multiple stages in the continuum of care [66].

To facilitate synthesis, the cost-effectiveness results were converted to US Dollars (USD) and inflated to 2012 prices [67,68]. The results are presented with summary information on the quality of the reporting, the costing perspective, and whether the health effects were measured in women or newborns, as these are important considerations for interpretation (Table 3). Meta-analysis was not appropriate given the diversity of strategies and differences in how the studies were framed. The country’s gross domestic product per capita (GDP-PC) (in 2012 prices) was used as a benchmark against which to consider the cost-effectiveness of strategies that reported the cost per life-year saved, cost per disability-adjusted life-year (DALY) averted or cost per quality-adjusted life-year (QALY) gained [69]. The World Health Organization (WHO) considers strategies and interventions to be cost-effective if the cost per DALY averted is less than three times the GDP-PC and highly cost-effective if less than the GDP-PC [70].

**Table 3:** **Cost**-**effectiveness results**

**Strategy**	**Comparator**	**Quality**	**Form of economic evaluation**	**Measured health effect in**	**Costing perspective**	**Used sensitivity analysis**	**CE result (US$ 2012)**	**CE measure**	GDP-PC (*)	**Reference**
**Cost per DALY averted**										
MNH services delivered at home, with community mobilization & HSS	Health system strengthening (HSS) at sub-district level	High	Field-based	Newborn	Societal (also reports programme)	Yes	126 (societal), 123 (programme)	per DALY averted	747	[22]
Home-based neonatal care by VHWs	No strategy	Low	Field-based	Newborn	Strategy	No	13	per DALY averted	1489	[39]
Home-based distribution of IPTp	IPTp distributed during ANC	High	Field-based	Newborn	Societal	Yes	3	per DALY averted	547	[59]
Train TBAs & supply clean delivery kits	No strategy	High	Economic Model (primary data)	Newborn	Societal	Yes	188 (project), 79 (10 year forecast)	per DALY averted	1469	[65]
Distribute malaria ITNs at ANC	No strategy	High	Economic Model (primary data)	Infant	Strategy	Yes	61	per DALY averted	272	[33]
Quality improvement collaborative	No strategy	High	Ec Model	Women	Health service provider	Yes	302	per DALY averted	383	[52]
Hospital-based promotion of breastfeeding	No strategy	High	Field-based	Newborn	Health service provider (excl. start-up)	No	164	per DALY averted	2264	[36]
**Cost per QALY gained**										
Outreach clinics by facility staff	Facility-based care	Medium	Field-based	Women	Health service provider & direct user (excl. start-up)	No	In site A/site B): 42/40 (S), 171/67 (C)	per QALY gained	747	[23]
Alternative delivery strategies FP & MCH	FP & MCH services provided at home by government fieldworkers	High	Field-based	Women	Health service provider	No	Range (low-high estimate): 87–139 (S1), 28–46 (S2), 68–109 (C)	per QALY gained	747	[25]
1. Community service points										
2. PHC										
**Cost per life-year saved (or year of life lost averted)**										
Women’s groups & HSS	HSS	Medium	Field-based	Newborn	Strategy	No	427 (trial), 284 (at scale)	per LYS	747	[18]
1. Women’s groups	No strategy	Medium	Field-based	Women & newborn	Strategy	No	149 (S1), 43 (S2)	per LYS	268	[49]
2. Peer counselling										
Women’s groups & HSS	HSS	High	Field-based	Newborn	Strategy	Yes	411, (489 incl. HSS)	per LYS	707	[51]
Women’s groups & HSS	HSS	Medium	Field-based	Newborn	Strategy	Yes	53, (77 incl. HSS)	per LYS	1489	[37]
Established Emergency Transport	No strategy	High	Field-based	Woman & newborn	Health service provider (excl. start-up)	Yes	21	per LYS	547	[61]
Outreach maternal health care	MH care at health post	High	Field-based	Woman & newborn	Societal	Yes	148-620	per LYS	512	[34]
Distribute malaria ITNs at ANC	No strategy	High	Economic model (using primary data)	Infant	Strategy	Yes	56	per LYS	272	[33]
HIV testing at ANC:	No strategy	High	Economic model	Newborn	Health service provider	Yes	80 (S1), 37 (S2)	per LYS	1489	[38]
1. nationwide,										
2. in high prevalence states										
**Cost per death averted(or cost per life saved)**										
Women’s groups & HSS	HSS	Medium	Field-based	Newborn	Strategy	No	13018 (trial), 8670 (at scale)	per death averted	747	[18]
Women’s groups & HSS	HSS	High	Field-based	Newborn	Strategy	Yes	11294, (13457 incl. HSS)	per death averted	707	[51]
Women’s groups & HSS	HSS	Medium	Field-based	Newborn	Strategy	Yes	1457, (2094 incl. HSS)	per death averted	1489	[37]
Women’s groups & Quality improvement at health facilities	No strategy	Low	Field-based	Newborn	Strategy	Yes	6138	per death averted	268	[47,48]
Home-based neonatal care by VHWs	No strategy	Low	Field-based	Newborn	Strategy	No	294	per death averted	1489	[39]
Home-based management of birth asphyxia by VHWs	Management of birth asphyxia by trained TBAs	Low	Field-based	Newborn	Equipment only	No	25	per death averted	1489	[40]
Train TBAs	No strategy	Low	Inference (from secondary data)	Newborn	Not specified	No	5744-13294	per death averted	747	[26]
Train TBAs & supply clean delivery kits	No strategy	High	Economic model (using primary data)	Newborn	Societal	Yes	4156 (trial), 1988 (10yr forecast)	per death averted	1469	[65]
MNH services delivered at home, with community mobilization & HSS	HSS at sub-district level	High	Field-based	Newborn	Societal (also reports programme)	Yes	3576 (societal), 3536 (programme)	per death averted	747	[22]
Tetanus toxoid (TT) immunization campaign	TT immunization at routine ANC	High	Field-based	Newborn	Strategy	Yes	1564 (S), 338–1438 (C)	per death averted	3557	[42]
Outreach maternal health care	MH care at health post	High	Field-based	Woman & newborn	Societal	Yes	1380-6414	per death averted	512	[34]
Distribute malaria ITNs at ANC	No strategy	High	Economic model (using primary data)	Infant	Strategy	Yes	1462	per death averted	272	[33]
Train midwives in newborn care	No strategy	Medium	Field-based	Newborn	Health service provider	No	402	per death averted	1469	[64]
Train new cadre in EmOC:	Obstetricians	High	Field-based	Newborn	Societal	Yes	14092 (CvS1), 3878 (CvS2), 240 (S2vS1)	per death averted	634	[28]
1. Medical doctors										
2. Clinical officers										
Hospital-based promotion of breastfeeding	Doing nothing	High	Field-based	Newborn	Health service provider (excl. start-up)	No	6894	per death averted	2264	[36]
Improved standard of special neonatal care	Doing nothing	Low	Field-based	Newborn	Equipment only	No	970	per death averted	2184	[56]
**Cost per strategy-specific measure**										
Community health education by midwives	No strategy	Low	Field-based	N/A	Strategy	No	5	per educational interaction	946	[32]
Promotion of NGO health clinics:	No strategy	High	Inference (from secondary data)	N/A	Strategy	Yes	<1 (S1), 15 (S2)	per additional ANC user	747	[21]
1. National media campaign										
2. National media campaign & local activities										
Establish community contact persons	No strategy	Low	Field-based	N/A	Strategy	No	259	per delivery with complications	1555	[54]
							7	per referral		
							37	per assisted delivery		
Home-based neonatal care by VHWs	No strategy	Low	Field-based	Newborn	Strategy	No	14	per home-visit for neonatal care	1489	[39]
Home-based neonatal care by VHWs	No strategy	Low	Field-based	N/A	Strategy	No	13	per home-visit for neonatal care	1489	[41]
Home-based distribution of IPTp	IPTp distributed during ANC	High	Field-based	Newborn	Societal	Yes	6 (S), 5 (C)	per women receiving full dose of IPTp	547	[59]
Tetanus toxoid (TT) immunization campaign	TT immunization at routine ANC	High	Field-based	Newborn	Strategy	Yes	20 (S), 7–30 (C)	per woman receiving full TT vaccine	3557	[42]
Vouchers for free MNH care, cash and in-kind transfers	No strategy	Medium	Field-based	N/A	Strategy	Yes	91	per additional delivery with qualified provider	747	[19]
Remove user fees for intrapartum care	No strategy	Medium	Field-based	N/A	Health service provider	No	3	per normal delivery	1032	[57]
							183	per C-section performed		
Established emergency transport scheme	No strategy	Low	Field-based	N/A	Strategy	No	44	per obstetric emergency transported	1555	[55]
HIV testing at ANC:	No strategy	High	Economic model (using secondary data)	Newborn	Health service provider	Yes	1060 (S1), 497 (S2)	per HIV infection prevented	1489	[38]
1. nationwide,										
2. in high prevalence states										
Strategies for abortion care:	No strategy	Medium	Economic Model (using secondary data)	N/A	Health service provider	No	135 (S1), 75 (S2), 102 (S3), 18 (S4)	per abortion case	547	[58]
1 Restricted-conventional										
2. Restricted-recommended										
3. Liberal-conventional										
4. Liberal-recommended										
Bamako Initiative	No strategy	Medium	Inference (from secondary data)	N/A	Health service provider	Yes	20 (Benin), 39 (Guinea)	per women receiving at least three antenatal visits	B: 752 G: 591	[27]
Quality improvement collaborative	No strategy	High	Economic Model	Women	Health service provider	Yes	155	per PPH averted,	383	[52]
							3	per delivery		
Distribute malaria ITN at ANC	No strategy	High	Field-based	N/A	Strategy	No	13	per ITN delivered to pregnant women	862	[43]
Introduce HIV testing:	No strategy	Medium	Field-based	N/A	Strategy	No	4 (S1), 4 (S2)	per person tested for HIV	946	[31]
1. at ANC										
2. at labour										
Syphilis testing at ANC	No strategy	Low	Field-based	Newborn	Strategy	No	6399	per adverse pregnancy outcome averted	1469	[63]
Decentralized programme of syphilis control	No strategy	Low	Field-based	Newborn	Strategy	No	293-346	per case of congenital syphilis averted	862	[45]
							114	per case of syphilis treated		
Decentralized programme of syphilis control	No strategy	Low	Field-based	Newborn	Strategy	No	252	per case of congenital syphilis averted	862	[46]
							137	per syphilis case treated		
Syphilis testing at ANC.	No strategy	Low	Field-based	N/A	Strategy	No	3 (S1), 11 (S2)	per person tested for syphilis	862	[44]
1. on-site										
2. standard clinics (off-site)										
Improve health and family welfare clinics	No strategy	Low	Inference (from primary and secondary data)	N/A	Strategy	No	14	per consultation	747	[20]
Initiative to promote facility-birth	No strategy	Medium	Field-based	N/A	Health service provider	Yes	1602, (201 excl. cost of strategy)	per facility-birth	634	[29]
Initiative to promote facility-birth	No strategy	Medium	Field-based	N/A	Societal	Yes	209	per facility-birth	634	[30]
Initiative on evidence-based practice in maternal and infant hospital care	No strategy	Medium	Field-based	N/A	Health service provider	No	49	cost saving per birth	3867	[62]
Hospital-based promotion of breastfeeding	No strategy	High	Field-based	Newborn	Health service provider (excl. start-up)	No	58	per neonatal case of diarrhoea averted	2264	[36]
							24	per birth		
Train Assistant Medical Officers in EmOC	Physicians	High	Field-based	N/A	Societal	Yes	61 (S),225 (C)	per C-section performed	579	[50]
Train new cadre in EmOC:	Obstetricians	High	sField-based	Newborn	Societal	Yes	248 (S1), 230 (S2), 615 (C)	per C-section performed	634	[28]
1. Medical doctors										
2. Clinical officers										
Programme on obstetric urogenital fistula	No strategy	Low	Field-based	N/A	Societal	No	1629-1745	per consultation	383	[53]

Note: multiple cost-effectiveness measures are reported for some strategies.

*Gross domestic product per capita (GDP-PC) in US$ 2012 prices is included as a benchmark against which to consider the cost per DALY averted, cost per QALY gained and cost per life-year saved. The WHO considers strategies and interventions to be cost-effective if the cost per DALY averted is less than three times the GDP-PC and highly cost-effective if less than the GDP-PC.

Acronyms used:

*ANC*: antenatal care; *C*: comparator; *CHW*: community health worker; *C*-section: caesarean section; *DALY*: disability-adjusted life-year; *EmOC*: emergency obstetric care; excl.: excluding; *FP*: family planning; *HSS*: health system strengthening; *Incl*.: including; *ITNs*: insecticide-treated bed nets; *IPTp*: intermittent preventive treatment in pregnancy; *MCH*: maternal and child health; *MNCH*: maternal, newborn, and child health; *MNH*: maternal and newborn health; *NGO*: non-governmental organisation; *QALY*: quality-adjusted life-year; *TBA*: traditional birth attendant; *PHC*: primary health care; *S*: strategy; *TT*: tetanus toxoid; *VHWs*: village health workers; *WHO*: World Health Organization.

## Results

The title and abstract of 3236 publications were screened and the full text of 140 publications was reviewed (Figure 1). This included three publications identified from reference lists [19,37,47]. One publication identified for full-text review could not be retrieved and so was not reviewed. Ninety-two publications were excluded at full-text review. Fourteen of the excluded publications were literature reviews, whose references were manually searched for relevant studies. One publication was excluded because the study was not in a LIC or LMIC. Sixteen publications were excluded because they assessed medical interventions rather than a strategy, and a further 6 publications were excluded because they focused on health topics, such as immunization or HIV, without specific reference to MNH care. Of those remaining, 8 publications were excluded because the cost-effectiveness of the strategy was not measured in a maternal or newborn population, though we did include one article that reported the cost per infant death averted. The latter reported on the distribution of mosquito nets to pregnant women and was included since the deaths averted would primarily be in newborns. A further 11 publications mentioned cost-effectiveness in the abstract but were excluded because they did not contain any cost data. Finally, 36 publications were excluded because they did not report a measure that combined cost and effect. For instance, several studies reported the total cost or a unit cost of implementing the strategy, such as the cost per health worker trained.

### Overview of the studies

**Figure 2:**
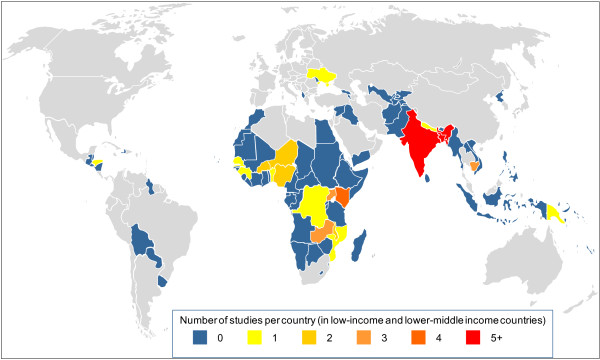
Geographic distribution of studies.

Of the 48 publications included in the review, 41 were articles from peer-reviewed journals and 7 were reports from the grey literature. Thirty-six were published since 2000, and 20 were published since 2009. The 48 publications reported on 43 separate studies undertaken in 21 different countries (Figure 2). Twenty-three studies were conducted in sub-Saharan Africa, 17 in Asia, and one each in Honduras, Papua New Guinea and Ukraine. Just over half (n = 23) of the studies were from five countries: Bangladesh (n = 8), India (n = 5), Kenya (n = 4), Uganda (n = 3) and Zambia (n = 3).

### Strategies to improve utilization and provision of MNH care

**Figure 3:**
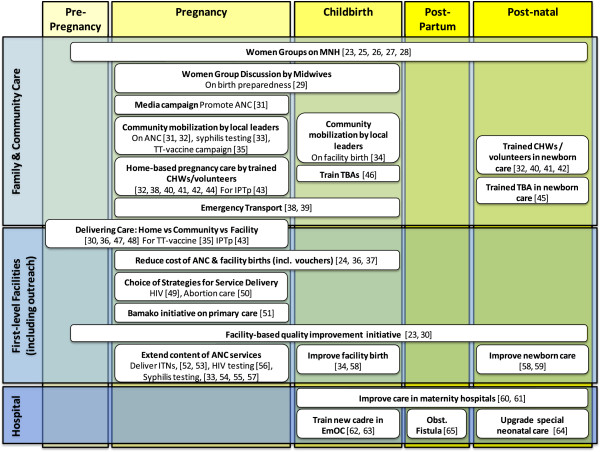
Innovations by place of care and lifecycle in the continuum of care.

The review identified a wide range of strategies to improve the utilization and provision of MNH care (Table 2). Some strategies focus on improving the coverage of a specific intervention or behaviour, while others cover multiple aspects of MNH care. The majority of the strategies focused on care during pregnancy and many involved community-based strategies, either to stimulate demand for MNH care or complement facility-based services. Although each strategy is distinct, there were some common themes, which we describe below and depict visually by presenting the different strategies in relation to the continuum of care and the different levels of the health system (Figure 3).

Health promotion and health education were central to many of the community-based strategies. Women’s groups were used to improve MNH in five related studies implemented in Bangladesh, India, Nepal, and Malawi [18,37,47,49,51]. Each study had a similar strategy, which involved training literate women from the local community to facilitate monthly women’s group meetings and work with participants to identify priority issues and implement local solutions, such as establishing a community-fund, stretcher schemes, and supplying clean delivery kits. Inspired by the use of women’s groups in Nepal, researchers in Cambodia piloted a participatory approach in which midwives held focus group discussions with pregnant women on birth preparedness and danger signs [32]. Community mobilization also featured in other strategies, and local leaders were used to help promote attendance at antenatal care (ANC) [21,22,52], syphilis testing [63], facility-births [29], and an immunization campaign [42].

Other strategies focused on removing barriers to care. Three reduced the cost of maternal care: user fees for intra-partum care were removed in Senegal to encourage women to deliver in facility, a nominal fixed charge was introduced for all maternal health services in The Gambia [34,57] and the third study combined vouchers for pregnant women to access free maternal care, cash to cover transport costs and in-kind items [19]. A further two studies evaluated emergency transport schemes, which had been established to facilitate referral for women with pregnancy or obstetric complications [55,61].

Various strategies were used to provide or improve MNH care at home and in the community. Several studies evaluated the cost-effectiveness of recruiting and training community health workers and volunteers to undertake home-based care [39-41,49,54,59]. Their role typically involved identifying pregnant women, providing advice on preparing for birth, on danger signs and on breastfeeding, however the extent of their responsibilities varied. In some instances, their role also included distribution of malaria prophylaxis, folic acid and iron supplements to pregnant women [59], or the management of birth asphyxia and treatment of neonatal sepsis [39-41]. Training traditional birth attendants (TBAs) was another strategy to improve community-based MNH care, though only one study was specifically designed to evaluate this [65], as estimates from Bangladesh were based on secondary sources [26].

The provision of family planning and MNH services by facility-based staff in outreach clinics was another theme [23,25,34,52]. Most of these studies compared the cost-effectiveness of alternative strategies for service delivery. For example, a tetanus toxoid campaign and home-based distribution of malaria prophylaxis were compared to routine antenatal care [42,59]. Economic models were also used to assess whether HIV screening in pregnancy should be restricted to high prevalence states [38] and to estimate the cost-effectiveness of four variants of abortion care [58].

Strategies to improve and extend facility-based services were also deployed. The strategies were wide-ranging though typically included training, equipment and supplies. Examples include quality improvement initiatives that encouraged health workers to identify and address problems with the care provided in their facility [47,52], and the Bamako Initiative on primary care, which was evaluated in Benin and Guinea using secondary data on the utilization of antenatal care [27]. Other studies focused on specific aspects of MNH care. Several strategies extended routine antenatal care, by distributing mosquito nets or introducing testing for HIV or syphilis [31,33,43-46,63]. The quality of intra-partum and post-natal care was a specific focus in some studies, both at primary facilities [20,29,64] and maternity hospitals [36,62]. In addition, two studies assessed the cost-effectiveness of training mid-level health workers to provide emergency obstetric care [28,50]. Finally, there were two studies focusing on specialist elements of MNH care: one on intensive and special neonatal care in Papua New Guinea [56] and the other on the provision of obstetric fistula care in Niger [53].

### Study design and evaluation

In the vast majority of studies, the strategy was compared to the situation before or without the strategy, though there were a few examples that compared the provision of MNH care at a facility with care at home, or in a community outreach clinic. Seven studies were conducted in the context of cluster randomized trials (including 2 with a factorial design), 3 were pre-post studies with a control, and 16 studies had a pre-post design without a control group. In addition, 7 studies compared intervention and control areas, 2 used prospective cohort data, 4 used economic modelling, and 4 used secondary data to estimate the cost-effectiveness results.

The effect of the strategies on the utilization and provision of health care and on health outcomes was measured using various indicators. Several studies evaluated the change in maternal or newborn mortality rates. Other studies report the effect on health care interactions or coverage of interventions, such as the percentage of pregnant women having at least three antenatal visits, the proportion of facility births or the number of pregnant women tested for HIV.

### Assessment of study quality

Sixteen studies were graded as high, 12 as medium, and 15 as low quality in their reporting (Table 3). Articles that reported cost-effectiveness results alongside other study outcomes tended to omit important methodological information. For example, several studies were not explicit about the costing perspective or the costs included. In comparison, articles that had cost-effectiveness as the primary objective were more comprehensive in their reporting. These articles usually provided detail on the rationale for the cost-effectiveness analysis and methods used, and many reported on sensitivity analyses that had been undertaken to explore uncertainty around the cost-effectiveness ratio. Most of the studies reported the incremental cost-effectiveness of a strategy (either compared to an alternative strategy or doing nothing) though four studies used average cost-effectiveness ratios to compare alternative strategies [23,25,42,50], which can be misleading and hide the true cost of achieving incremental health care goals [71].

There were large differences in the quality and content of the economic evaluations conducted, particularly in the approach and methods used to estimate costs. The majority of studies adopted a health service provider perspective, and relatively few took into account the costs incurred by households to access MNH care. Some studies analysed only recurrent costs, having excluded any initial set-up costs [23,36,46,61], and several analysed the cost of the strategy without taking into account the cost implications of delivering maternal and newborn care [18-21,32,37,39,41,47,49,51,54,55]. For example, use of women’s groups resulted in increases in the uptake of antenatal care in Malawi and Nepal and institutional deliveries in Nepal, but the additional cost of this increase in service utilization was not taken into account [14]. In addition, a couple of studies included only the cost of equipment and supplies [40,56]. In contrast, a number of studies were more comprehensive and used economic rather than financial costing, which meant market values were included for donated goods and volunteer time [22,33,34,50,52,53,59,65].

### Evidence of cost-effectiveness

A range of measures were used to report the cost-effectiveness of strategies to improve the utilization and provision of MNH care, and many studies reported more than one outcome (Table 3). The most common single measure was the cost per life saved (also known as cost per death averted) and this was used in 16 of the 43 studies. Thirteen of these studies focused on newborns, one on infants [33] and two estimated lives saved in both women and newborns [34]. Seven studies reported the cost per life-year saved (or cost per year of life lost averted) in either women or their newborns [33,34,38,49,51,61]. This measure takes into account not only the number of lives saved but also the number of years of life, based on the beneficiary’s age and their expected length of life. There were also nine cost-utility studies, where the effect on health is measured in a composite indicator combining the number of years of life and the quality of life: seven reported the cost per DALY averted [22,33,36,39,52,59,65] and two reported the cost per QALY gained [23,25]. Twenty studies reported strategy-specific cost-effectiveness measures. Some referred to a specific health effect, such as case of congenital syphilis averted, while others reported on the health care received, using measures such as the cost per insecticide-treated mosquito net (ITN) delivered, cost per facility-birth, or cost per home visit.

In the vast majority of studies, the strategy was found to be more effective but also more costly than its comparator, and therefore the decision on whether to adopt a strategy depends on the decision maker’s willingness to pay for improvements in health or health care. An exception was in Ukraine where efforts to encourage evidence-based policies and reduce the use of Caesarean sections was found to be cost saving. There was also evidence that training mid-level cadres in emergency obstetric care had a lower average cost per life saved than training obstetricians [28]. Using GDP per capita as a benchmark against which to consider the cost per DALY averted, cost per QALY gained and cost per life-year saved, all the strategies that report these measures would be considered cost-effective [70].

## Discussion

Our systematic review identified 48 publications on the cost-effectiveness of strategies to improve the utilization and provision of MNH care in low-income and lower-middle-income countries. The 48 publications reported on 43 separate studies and we judged the reporting of 16 of these studies to be of high quality with respect to the CHEERS reporting criteria.

There was considerable diversity in the strategies used to improve MNH care, and also in the setting, intensity and scale of implementation. However, it was possible to identify some common themes among the strategies, and these were presented in relation to the continuum of care and the level of the health system. This synthesis summarized the cost-effectiveness literature available and highlights the extent to which the evidence focuses on community-based strategies and care during pregnancy. It also emphasizes the lack of evidence on the cost-effectiveness of strategies to improve post-partum care. In addition, it was interesting to note the large proportion of strategies focused on a specific aspect of MNH care. This was particularly noticeable in the facility-based strategies, which included a number of strategies to extend antenatal care and improve specialist care.

It is clear that demand and supply-side strategies can be cost-effective in enhancing the utilization and provision of MNH care and improving health outcomes. Strategies that reported on the cost per life-year saved and cost per DALY averted were cost-effective when compared to GDP per capita. These strategies, in their specific settings, included the use of women’s groups [18,37,47,49,51]; home-based newborn care using community health workers, volunteers and traditional birth attendants [22,39,59,65]; adding services to routine antenatal care [33]; a facility-based quality improvement initiative to enhance compliance to care standards [52]; and the promotion of breastfeeding in maternity hospitals [36]. However, it should be noted that the results may not be transferable beyond the study setting and using GDP per capita as a threshold does not necessarily ensure that the strategy is affordable. Using GDP per capita as the threshold also approaches cost-effectiveness from a national perspective, and it has been suggested that a global minimum monetary value for a DALY would improve the transparency and efficiency of priority setting for international donors [72].

It is more difficult to interpret the cost-effectiveness results when strategy-specific measures were reported, such as the cost per Caesarean-section, or cost per syphilis case treated. Very few studies use the same measure, though some report estimates from the same setting at different points in time [39,41,44-46]. In many of the studies the costs appear low given the potential health benefits, though the results should be interpreted with care as the choice of measure can also affect conclusions. For example, the cost per woman vaccinated with tetanus toxoid was much lower when the vaccination took place in a campaign rather than during routine antenatal care, however, targeting the vaccine to pregnant women attending antenatal care was found to be more cost-effective when the cost per life saved was estimated [42]. This example highlights the benefits of using health outcomes data, and where that is not available, the value in extrapolating beyond intermediate measures to estimate the number of lives saved using models, such as the Lives Saved Tool (LiST) [73]. Strategy-specific measures also have limited use for priority-setting as decision-makers cannot directly compare strategies that report different cost-effectiveness measures.

The extent to which alternative strategies can be compared was also limited by the how the cost-effectiveness analysis was framed. The reported results may depend on the choice of comparator, the costs included and any assumptions about the effect of the strategy on the quantity and quality of life. For instance, a commentary accompanying the article on training assistant medical officers to perform emergency obstetric care argued the cost-effectiveness results may be an under-estimate since ‘doing nothing’ would be a more realistic comparator than training surgeons given the shortage of medical personnel in Mozambique [50]. The costing perspective is another dimension that can affect study results. For instance, from a health services perspective, delivering home-based care was found to be more expensive than providing services in an outreach clinic or at the health facility, but this perspective does not take into account the direct and indirect costs incurred by households to obtain care [25]. The range of costs included can also have a dramatic impact. For example, when interpreting the results of the study on the management of birth asphyxia by health volunteers it is important to acknowledge the estimate of US$25 per life saved only takes into account the cost of the equipment, and does not include the cost of other supplies or value the time donated by health volunteers [40].

We had hoped to learn the extent to which cost-effectiveness depends on the strength or scale of implementation, or country-specific factors, and each of these considerations is salient for the transferability of study results. Intensive implementation may improve the strategy’s effectiveness, but this is likely to come at a cost. Similarly, the scale of implementation may affect costs or effects, and the potential to generate economies of scale will depend on the characteristics of the strategy and the geographic setting. The context for a study can also influence costs and effects, and the degree to which a strategy can be replicated in another setting. One study reported that there would be economies of scale if the women’s group initiative were rolled out in Bangladesh, and another on the Bamako Initiative reported relatively similar cost-effectiveness in Benin and Guinea [18,27]. However, drawing conclusions across studies is extremely challenging given the wide-ranging strategies implemented in disparate contexts. The women’s group studies were the most comparable since a common strategy was applied in different countries and with differing intensity (in terms of population coverage), and they were methodologically similar as the studies had several researchers in common. Looking across these studies there was some evidence that greater intensity of implementation produced a larger effect, though implementation and contextual factors may impact on the effect sizes [14]. As others have noted, understanding determinants of differences in cost, and the effect of scale on cost are priorities for future work [14].

Only three studies reported a cost-effectiveness measure that took into account the combined effect of the strategy on both maternal and newborn health. One of the distinctive characteristics of pregnancy and intra-partum care is the potential for the care to benefit both the woman and her child. In addition, intervening during pregnancy can affect subsequent care seeking and health outcomes. Thus, the cost-effectiveness of a strategy may be under-estimated if the health benefits were measured in either women or newborns but not both [74], though this point was not highlighted in any of the publications.

In drawing conclusions we need to bear in mind the quality of the publications. The CHEERS checklist sets a standard for the information that should be reported, and although there were some high quality publications, many fell short of the standard. The publications of low and medium quality were often those in which the cost-effectiveness analysis was presented as supplementary to other study results and a strategy-specific measure was used. In some cases, gaps in reporting made it difficult to assess whether methods were appropriate, what assumptions had been made and how the findings should be interpreted. As many peer-reviewed journals have restrictions on article length, authors should make better use of web appendices or, ideally, report the cost-effectiveness analysis as a separate, stand-alone article. Discussion of the cost-effectiveness results was another aspect where the quality was often limited, and conclusions on the cost-effectiveness of the strategy were often stated with only a cursory consideration of study design, context, and limitations, and without reference to the existing literature.

However, the checklist also has some limitations. It focuses on the quality of the reporting rather than the quality of the economic evaluation conducted. This means articles with a clear description of their methods may rank highly even when there are shortcomings in how the study was framed or the range of costs included. In addition, several of the criteria more readily apply to cost-effectiveness analyses that use individual patient-level data or economic modelling, which are the backbone of cost-effectiveness studies in high income countries, but less frequently used in low and middle income countries. We also acknowledge the quality categorization was based on a simple approach and alternative methodologies could be applied, such as assigning weights to the criteria based on subjective judgment or statistical analysis.

We took a systematic approach to the literature search and selection process, though there is always a risk that a relevant article has been missed, and it was not always straightforward to determine whether an article satisfied the inclusion criteria. For example, we carefully considered whether to include article publication that compared universal HIV screening in pregnant women with screening restricted to areas of high HIV prevalence [38]. We also carefully considered the eligibility of several articles that reported costs in relation to a process indicator, such as the study from Cambodia on midwife-led group discussions with pregnant women that reported the cost per educational interaction [32]. We decided that process indicators would be eligible if they referred to an interaction between women (or newborns) and a front-line worker. Publication bias is also a potential concern, since cost-effectiveness analyses are often only undertaken in field-studies once a positive effect has been demonstrated, though the lack of statistically significant effect does not necessarily preclude a strategy from being cost-effective, nor should it prevent a cost-effectiveness analysis being published [75,76]. That all the studies in our review had positive findings was striking, and suggests that cost-effectiveness analyses with negative findings may not have been published.

The review highlights a need for future research. Only a minority of studies on strategies to improve the utilization and provision of MNH care consider their cost-effectiveness [8-14]. Moreover, the evidence available is limited by the lack of high quality reports presenting comparable cost-effectiveness measures. In particular, there are gaps relating to post-partum and post-natal care, and relatively few studies focused on the quality of intra-partum care. Further work on the extent to which implementation, scale and context impact cost-effectiveness would also be useful to understand the degree to which strategies can be replicated elsewhere. Moreover, for future research to generate results that can be transferred beyond the immediate study setting, greater consideration should be given to how the economic evaluation is framed. This includes the use of a comparable cost-effectiveness measure, such as cost per DALY averted, and also a costing that reflects the full cost to everyone of implementing the strategy within the prevailing health system. It is good practice to take household costs into account and to value donated goods and volunteer time. As the initiative to promote facility-births in Burkina Faso demonstrated, the cost of the strategy (as opposed to the intervention alone) can be substantial: there was an eightfold increase in the cost of a facility birth when programme costs were included in the calculations [29]. Thus, estimates of the cost-effectiveness of life-saving MNH interventions that do not take into account demand or supply strategies will substantially under-estimate the resources required to reduce maternal and neonatal mortality.

## Conclusion

Demand and supply-side strategies can be cost-effective in enhancing the utilization and provision of MNH care and improving health outcomes, though the evidence available is limited by the lack of high quality studies using comparable cost-effectiveness measures. Direct comparison of alternative strategies was also limited by how the studies were framed, as there was substantial variation in how researchers approached, designed and analyzed cost-effectiveness. Further studies are needed, though existing evidence shows the cost-effectiveness of several strategies implemented in the community to influence health practices and care seeking, and also facility-based initiatives to improve the range and quality of MNH care available.

## Abbreviations

Adj: Adjusted; ANC: Antenatal care; C: Comparator; CHEERS: Consolidated Health Economic Evaluation Reporting Standards; CHW: Community health worker; CI: Confidence interval; C-section: Caesarean section; DALY: Disability-adjusted life year; EmOC: Emergency obstetric care; Excl: Excluding; FP: Family planning; FWA: Family welfare assistant; GDP-PC: Gross domestic product per capita; HIV: Human immunodeficiency virus; HSS: Health system strengthening; IMR: Infant mortality rate; Incl: Including; ITNs: Insecticide-treated bed nets; IPTp: Intermittent preventive treatment in pregnancy; LB: Live births; LBW: Low birth weight; LIC: Low-income country; LiST: Lives Saved Tool; LMIC: Lower-middle income country; MCH: Maternal and child health; MNCH: Maternal, newborn, and child health; MNH: Maternal and newborn health; MMR: Maternal mortality rate; NGO: Non-governmental organisation; NHS: National Health Service; NMR: Neonatal mortality rate; OR: Odds ratio; PHC: Primary health care; QALY: Quality-adjusted life-year; RCT: Randomized control trial; RR: Relative risk; S: Strategy; S1: Strategy 1; S2: Strategy 2; TBA: Traditional birth attendant; TT: Tetanus toxoid; USD: United States Dollars; VHW: Village health workers; WHO: World Health Organization.

## Competing interests

The authors declare that they have no competing interests.

## Authors’ contributions

LMJ prepared a protocol for the systematic review with advice from JS, AM, SC and CP. LMJ registered the review. LMJ and CP independently conducted the title and abstract review and the full-text review. LMJ led the data extraction, which was checked by CP. LMJ and CP assessed study quality. LMJ synthesized the findings and drafted the manuscript, with assistance from CP, AM, JS and SC. All authors approved the final manuscript.

## Pre-publication history

The pre-publication history for this paper can be accessed here:

http://www.biomedcentral.com/1471-2393/14/243/prepub

## Supplementary Material

Additional file 1 Search Strategy. Click here for file

Additional file 2 List of eligible countries. Click here for file
