# Erratum: Cost-effectiveness of strategies to improve the utilization and provision of maternal and newborn health care in low-income and lower-middle-income countries: a systematic review

**DOI:** 10.1186/s12884-015-0476-5

**Published:** 2015-03-21

**Authors:** Lindsay Mangham-Jefferies, Catherine Pitt, Simon Cousens, Anne Mills, Joanna Schellenberg

**Affiliations:** Department of Global Health and Development, London School of Hygiene and Tropical Medicine, London, UK; Department of Infectious Disease Epidemiology, London School of Hygiene and Tropical Medicine, London, UK; Department of Disease Control, London School of Hygiene and Tropical Medicine, London, UK

## Erratum

During the publication process of this article [1] a number of errors were unfortunately introduced into the references included in figure three, and were not noticed until after the final version had been published. The corrected version can be seen here (Figure 1).

**Figure 1:**
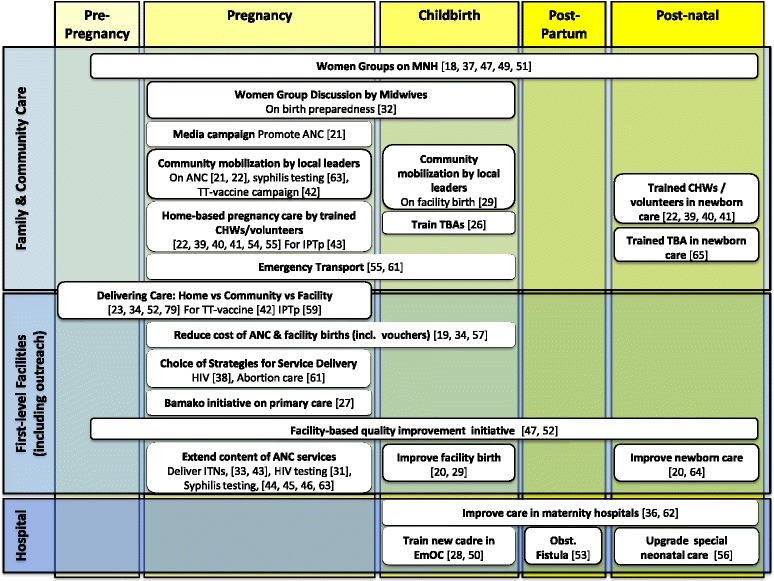
**Innovations by place of care and lifecycle in the continuum of care.**
