# Supplementary material for: Cost-effectiveness of strategies to improve the utilization and provision of maternal and newborn health care in low-income and lower-middle-income countries: a systematic review
Source: BMC Pregnancy Childbirth. 2014 Jul 22;14:243. doi: 10.1186/1471-2393-14-243 (PMC4223592; doi:10.1186/1471-2393-14-243)
Supplement: Additional file 1 — Search Strategy. [file 1471-2393-14-243-S1.pdf]

Additional File A: Search Strategy

MEDLINE

#1 economics/ or exp costs and cost analysis/ or exp economics, hospital/ or economics, medical/

#2 (economic$ or cost$ or efficien$ or pric$ or budget$).ti,ab.

#3 (value adj2 money).ti,ab.

#4 (expenditure$ not energy).ti,ab.

#5 1 or 2 or 3 or 4

#6 ((energy or oxygen or metabolic) adj (cost or expenditure)).ti,ab.

#7 5 not 6

#8 maternal health services/ or prenatal care/ or perinatal care/ or delivery, obstetric/ or postnatal care/

#9 (maternal or maternity or mother or neonatal or newborn or pregnancy or prenatal or antenatal or ante natal or intrapartum or intra partum or birth or childbirth or obstetric or postpartum or post partum or perinatal or peri natal or postnatal or post natal) ADJ5 (health or care or service$ or facility$ or visit$ or interaction$ or contact$ or communicat$ or relation$).ti,ab.

#10 8 or 9

#11 (Africa or Asia or Caribbean or West Indies or South America or Latin America or Central America or Middle East).**ti,ab,kw.**

#12 (Afghanistan or Albania or Armenia or Armenian or Bangladesh or Benin or Belize or Bhutan or Bolivia or Burkina Faso or Burkina Fasso or Upper Volta or Burundi or Urundi or Cambodia or Khmer Republic or Kampuchea or Cameroon or Cameroons or Cameron or Camerons or Cameroun or Cape Verde or Central African Republic or Chad or Comoros or Comoro Islands or Mayotte or Congo or Zaire or Cote d'Ivoire or Ivory Coast or Djibouti or French Somaliland or East Timor or East Timur or Timor Leste or Egypt or United Arab Republic or El Salvador or Eritrea or Ethiopia or Fiji or Gambia or Gaza or Georgia or Georgian or Ghana or Gold Coast or Guatemala or Guinea or Guam or Guiana or Guyana or Haiti or Honduras or India or Indonesia or Iraq or Kenya or Kiribati or Korea or Kosovo or Kyrgyzstan or Kirghizia or Kyrgyz Republic or Kirghiz or Kirgizstan or Lao PDR or Laos or Lesotho or Basutoland or Liberia)**. ti,ab,kw.**

#13 (Madagascar or Malagasy Republic or Malawi or Nyasaland or Mali or Marshall Islands or Mauritania or Agalega Islands or Micronesia or Moldova or Moldovia or Moldovian or

Morocco or Ifni or Mozambique or Myanmar or Myanma or Burma or Nepal or Netherlands Antilles or New Caledonia or Nicaragua or Niger or Nigeria or Pakistan or Palestine or Paraguay or Philippines or Philipines or Phillipines or Phillippines or Rwanda or Ruanda or Samoa or Samoan Islands or Navigator Island or Navigator Islands or Sao Tome or Senegal or Sierra Leone or Sri Lanka or Ceylon or Solomon Islands or Somalia or Sudan or Swaziland or Syria or Tajikistan or Tadzhikistan or Tadjikistan or Tadzhik or Tanzania or Togo or Togolese Republic or Tonga or Trinidad or Tobago or Tunisia or Turkey or Turkmenistan or Turkmen or Uganda or Ukraine or Uzbekistan or Uzbek or Vanuatu or New Hebrides or Vietnam or Viet Nam or West Bank or Yemen or Zambia or Zimbabwe or Rhodesia). **ti,ab,kw.**

#14 (developing or less developed or under developed or underdeveloped or middle income or low income) ADJ (countr$ or nation$ or population$ or world or econom$).**ti,ab,kw.**

#15 low$ ADJ (gdp or gnp or gross domestic or gross national).**ti,ab,kw.**

#16 11 or 12 or 13 or 14 or 15

#17 7 and 10 and 16

#18 (letter or editorial or historical article).pt.

#19 17 not 18

#20 limit 19 to humans

#21 limit 20 to yr="1990 -Current"

EMBASE

#1 health-economics/ or exp economic-evaluation/ or exp health-care-cost/

#2 (economic$ or cost$ or efficien$ or pric$ or budget$).ti,ab.

#3 (value adj2 money).ti,ab.

#4 (expenditure$ not energy).ti,ab.

#5 1 or 2 or 3 or 4

#6 ((energy or oxygen or metabolic) adj (cost or expenditure)).ti,ab.

#7 5 not 6

#8 maternal care/ or prenatal care/ or perinatal care/ or delivery/ or postnatal care/

#9 (maternal or maternity or mother or neonatal or newborn or pregnancy or prenatal or antenatal or ante natal or intrapartum or intra partum or birth or childbirth or obstetric or postpartum or post partum or perinatal or peri natal or postnatal or post natal) ADJ5 (health or care or service$ or facility$ or visit$ or interaction$ or contact$ or communicat$ or relation$).ti,ab.

#10 8 or 9

#11 (Africa or Asia or Caribbean or West Indies or South America or Latin America or Central America or Middle East).**ti,ab,kw.**

#12 (Afghanistan or Albania or Armenia or Armenian or Bangladesh or Benin or Belize or Bhutan or Bolivia or Burkina Faso or Burkina Fasso or Upper Volta or Burundi or Urundi or Cambodia or Khmer Republic or Kampuchea or Cameroon or Cameroons or Cameron or Camerons or Cameroun or Cape Verde or Central African Republic or Chad or Comoros or Comoro Islands or Mayotte or Congo or Zaire or Cote d'Ivoire or Ivory Coast or Djibouti or French Somaliland or East Timor or East Timur or Timor Leste or Egypt or United Arab Republic or El Salvador or Eritrea or Ethiopia or Fiji or Gambia or Gaza or Georgia or Georgian or Ghana or Gold Coast or Guatemala or Guinea or Guam or Guiana or Guyana or Haiti or Honduras or India or Indonesia or Iraq or Kenya or Kiribati or Korea or Kosovo or Kyrgyzstan or Kirghizia or Kyrgyz Republic or Kirghiz or Kirgizstan or Lao PDR or Laos or Lesotho or Basutoland or Liberia)**. ti,ab,kw.**

#13 (Madagascar or Malagasy Republic or Malawi or Nyasaland or Mali or Marshall Islands or Mauritania or Agalega Islands or Micronesia or Moldova or Moldovia or Moldovian or Morocco or Ifni or Mozambique or Myanmar or Myanma or Burma or Nepal or Netherlands Antilles or New Caledonia or Nicaragua or Niger or Nigeria or Pakistan or Palestine or Paraguay or Philippines or Philipines or Phillipines or Phillippines or Rwanda or Ruanda or Samoa or Samoan Islands or Navigator Island or Navigator Islands or Sao Tome or Senegal or Sierra Leone or Sri Lanka or Ceylon or Solomon Islands or Somalia or Sudan or Swaziland or Syria or Tajikistan or Tadzhikistan or Tadjikistan or Tadzhik or Tanzania or Togo or Togolese Republic or Tonga or Trinidad or Tobago or Tunisia or Turkey or Turkmenistan or Turkmen or Uganda or Ukraine or Uzbekistan or Uzbek or Vanuatu or New Hebrides or Vietnam or Viet Nam or West Bank or Yemen or Zambia or Zimbabwe or Rhodesia). **ti,ab,kw.**

#14 (developing or less developed or under developed or underdeveloped or middle income or low income) ADJ (countr$ or nation$ or population$ or world or econom$).**ti,ab,kw.**

#15 low$ ADJ (gdp or gnp or gross domestic or gross national).**ti,ab,kw.**

#16 11 or 12 or 13 or 14 or 15

#17 7 and 10 and 16

#18 (letter or editorial or historical article).pt.

#19 17 not 18

#20 limit 19 to humans

#21 limit 20 to yr="1990 -Current"

GLOBAL HEALTH

#1 economics/ or exp economic evaluation/ or costs/ or health care costs/ or cost analysis/ or cost benefit analysis/ or cost effectiveness analysis/

#2 (economic$ or cost$ or efficien$ or pric$ or budget$).ti,ab.

#3 (value adj2 money).ti,ab.

#4 (expenditure$ not energy).ti,ab.

#5 1 or 2 or 3 or 4

#6 ((energy or oxygen or metabolic) adj (cost or expenditure)).ti,ab.

#7 5 not 6

#8 maternity services/ or prenatal care/ or obstetrics/

#9 (maternal or maternity or mother or neonatal or newborn or pregnancy or prenatal or antenatal or ante natal or intrapartum or intra partum or birth or childbirth or obstetric or postpartum or post partum or perinatal or peri natal or postnatal or post natal) ADJ5 (health or care or service$ or facility$ or visit$ or interaction$ or contact$ or communicat$ or relation$).ti,ab.

#10 8 or 9

#11 (Africa or Asia or Caribbean or West Indies or South America or Latin America or Central America or Middle East).**ti,ab.**

#12 (Afghanistan or Albania or Armenia or Armenian or Bangladesh or Benin or Belize or Bhutan or Bolivia or Burkina Faso or Burkina Fasso or Upper Volta or Burundi or Urundi or Cambodia or Khmer Republic or Kampuchea or Cameroon or Cameroons or Cameron or Camerons or Cameroun or Cape Verde or Central African Republic or Chad or Comoros or Comoro Islands or Mayotte or Congo or Zaire or Cote d'Ivoire or Ivory Coast or Djibouti or French Somaliland or East Timor or East Timur or Timor Leste or Egypt or United Arab Republic or El Salvador or Eritrea or Ethiopia or Fiji or Gambia or Gaza or Georgia or Georgian or Ghana or Gold Coast or Guatemala or Guinea or Guam or Guiana or Guyana or Haiti or Honduras or India or Indonesia or Iraq or Kenya or Kiribati or Korea or Kosovo or Kyrgyzstan or Kirghizia or Kyrgyz Republic or Kirghiz or Kirgizstan or Lao PDR or Laos or Lesotho or Basutoland or Liberia)**. ti,ab.**

#13 (Madagascar or Malagasy Republic or Malawi or Nyasaland or Mali or Marshall Islands or Mauritania or Agalega Islands or Micronesia or Moldova or Moldovia or Moldovian or Morocco or Ifni or Mozambique or Myanmar or Myanma or Burma or Nepal or Netherlands Antilles or New Caledonia or Nicaragua or Niger or Nigeria or Pakistan or Palestine or Paraguay or Philippines or Philipines or Phillipines or Phillippines or Rwanda or Ruanda or Samoa or Samoan Islands or Navigator Island or Navigator Islands or Sao Tome or Senegal or Sierra Leone or Sri Lanka or Ceylon or Solomon Islands or Somalia or Sudan or Swaziland or

Syria or Tajikistan or Tadzhikistan or Tadjikistan or Tadzhik or Tanzania or Togo or Togolese Republic or Tonga or Trinidad or Tobago or Tunisia or Turkey or Turkmenistan or Turkmen or Uganda or Ukraine or Uzbekistan or Uzbek or Vanuatu or New Hebrides or Vietnam or Viet Nam or West Bank or Yemen or Zambia or Zimbabwe or Rhodesia). **ti,ab.**

#14 (developing or less developed or under developed or underdeveloped or middle income or low income) ADJ (countr$ or nation$ or population$ or world or econom$).**ti,ab.**

#15 low$ ADJ (gdp or gnp or gross domestic or gross national).**ti,ab.**

#16 11 or 12 or 13 or 14 or 15

#17 7 and 10 and 16

#18 (letter or editorial or historical article).pt.

#19 17 not 18

#20 limit 19 to yr="1990 -Current"

ECONLIT

#1 (economic$ or cost$ or efficien$ or pric$ or budget$ or value for money).ti,ab.

#2 (value adj2 money).ti,ab.

#3 (expenditure$ not energy).ti,ab.

#4 1 or 2 or 3

#5 ((energy or oxygen or metabolic) adj (cost or expenditure)).ti,ab.

#6 4 not 5

#7 (maternal or maternity or mother or neonatal or newborn or pregnancy or prenatal or antenatal or ante natal or intrapartum or intra partum or birth or childbirth or obstetric or postpartum or post partum or perinatal or peri natal or postnatal or post natal) ADJ5 (health or care or service$ or facility$ or visit$ or interaction$ or contact$ or communicat$ or relation$).ti,ab.

#8 (Africa or Asia or Caribbean or West Indies or South America or Latin America or Central America or Middle East).**ti,ab,kw.**

#9 (Afghanistan or Albania or Armenia or Armenian or Bangladesh or Benin or Belize or Bhutan or Bolivia or Burkina Faso or Burkina Fasso or Upper Volta or Burundi or Urundi or Cambodia or Khmer Republic or Kampuchea or Cameroon or Cameroons or Cameron or Camerons or Cameroun or Cape Verde or Central African Republic or Chad or Comoros or Comoro Islands or Mayotte or Congo or Zaire or Cote d'Ivoire or or Ivory Coast or Djibouti or French Somaliland or East Timor or East Timur or Timor Leste or Egypt or United Arab Republic or El

Salvador or Eritrea or Ethiopia or Fiji or Gambia or Gaza or Georgia or Georgian or Ghana or Gold Coast or Guatemala or Guinea or Guam or Guiana or Guyana or Haiti or Honduras or India or Indonesia or Iraq or Kenya or Kiribati or Korea or Kosovo or Kyrgyzstan or Kirghizia or Kyrgyz Republic or Kirghiz or Kirgizstan or Lao PDR or Laos or Lesotho or Basutoland or Liberia)**. ti,ab,kw.**

#10 (Madagascar or Malagasy Republic or Malawi or Nyasaland or Mali or Marshall Islands or Mauritania or Agalega Islands or Micronesia or Moldova or Moldovia or Moldovian or Morocco or Ifni or Mozambique or Myanmar or Myanma or Burma or Nepal or Netherlands Antilles or New Caledonia or Nicaragua or Niger or Nigeria or Pakistan or Palestine or Paraguay or Philippines or Philipines or Phillipines or Phillippines or Rwanda or Ruanda or Samoa or Samoan Islands or Navigator Island or Navigator Islands or Sao Tome or Senegal or Sierra Leone or Sri Lanka or Ceylon or Solomon Islands or Somalia or Sudan or Swaziland or Syria or Tajikistan or Tadzhikistan or Tadjikistan or Tadzhik or Tanzania or Togo or Togolese Republic or Tonga or Trinidad or Tobago or Tunisia or Turkey or Turkmenistan or Turkmen or Uganda or Ukraine or Uzbekistan or Uzbek or Vanuatu or New Hebrides or Vietnam or Viet Nam or West Bank or Yemen or Zambia or Zimbabwe or Rhodesia). **ti,ab,kw.**

#11 (developing or less developed or under developed or underdeveloped or middle income or low income) ADJ (countr$ or nation$ or population$ or world or econom$).**ti,ab,kw.**

#12 low$ ADJ (gdp or gnp or gross domestic or gross national).**ti,ab,kw.**

#13 8 or 9 or 10 or 11 or 12

#14 6 and 7 and 13

#15 limit 14 to yr="1990 -Current"

WEB OF SCIENCE

#1 TS=(economic* or cost* or efficien* or pric* or budget*)

#2 TS=(value near/2 money)

#3 TS= (expenditure* not energy)

#4 #1 or #2 or #3

#5 TS= ((energy or oxygen or metabolic) near/1 (cost or expenditure))

#6 #4 not #5

#7 TS=((maternal or maternity or mother or neonatal or newborn or pregnancy or prenatal or antenatal or “ante natal” or intrapartum or “intra partum” or birth or childbirth or obstetric or postpartum or “post partum” or perinatal or “peri natal” or postnatal or “post natal”) NEAR/5 (health or care or service* or facility* or visit* or interaction* or contact* or communicat* or relation*))

#8 TS= (Africa or Asia or Caribbean or “West Indies” or “South America” or “Latin America” or “Central America” or “Middle East”)

#9 TS= (Afghanistan or Albania or Armenia or Armenian or Bangladesh or Benin or Belize or Bhutan or Bolivia or “Burkina Faso” or “Burkina Fasso” or “Upper Volta” or Burundi or Urundi or Cambodia or “Khmer Republic” or Kampuchea or Cameroon or Cameroons or Cameron or Camerons or Cameroun or “Cape Verde” or “Central African Republic” or Chad or Comoros or “Comoro Islands” or Mayotte or Congo or Zaire or “Cote d'Ivoire” or “Ivory Coast” or Djibouti or “French Somaliland” or “East Timor” or “East Timur” or “Timor Leste” or Egypt or “United Arab Republic” or “El Salvador” or Eritrea or Ethiopia or Fiji or Gambia or Gaza or Georgia or Georgian or Ghana or Gold Coast or Guatemala or Guinea or Guam or Guiana or Guyana or Haiti or Honduras or India or Indonesia or Iraq or Kenya or Kiribati or Korea or Kosovo or Kyrgyzstan or Kirghizia or “Kyrgyz Republic” or Kirghiz or Kirgizstan or “Lao PDR” or Laos or Lesotho or Basutoland or Liberia)

#10 TS=(Madagascar or “Malagasy Republic” or Malawi or Nyasaland or Mali or “Marshall Islands” or Mauritania or “Agalega Islands” or Micronesia or Moldova or Moldovia or Moldovian or Morocco or Ifni or Mozambique or Myanmar or Myanma or Burma or Nepal or “Netherlands Antilles” or “New Caledonia” or Nicaragua or Niger or Nigeria or Pakistan or Palestine or Paraguay or Philippines or Philipines or Phillipines or Phillippines or Rwanda or Ruanda or Samoa or “Samoan Islands” or “Navigator Island” or “Navigator Islands” or “Sao Tome” or Senegal or “Sierra Leone” or “Sri Lanka” or Ceylon or “Solomon Islands” or Somalia or Sudan or Swaziland or Syria or Tajikistan or Tadzhikistan or Tadjikistan or Tadzhik or Tanzania or Togo or “Togolese Republic” or Tonga or Trinidad or Tobago or Tunisia or Turkey or Turkmenistan or Turkmen or Uganda or Ukraine or Uzbekistan or Uzbek or Vanuatu or “New Hebrides” or Vietnam or “Viet Nam” or “West Bank” or Yemen or Zambia or Zimbabwe or Rhodesia)

#11 TS=((developing or “less developed” or “under developed” or underdeveloped or “middle income” or “low income”) NEAR/1 (countr* or nation* or population* or world or econom*))

#12 TS=((low*) NEAR/1 (gdp or gnp or “gross domestic” or “gross national”))

#13 #8 or #9 or #10 or #11 or #12

#14 #6 and #7 and #13

NHS EED Database

#1 maternal health services/ or prenatal care/ or perinatal care/ or delivery, obstetric/ or postnatal care/ IN NHSEED

#2 (maternal) OR (maternity) OR (mother) OR (newborn) OR (neonatal) OR (pregnancy) OR (prenatal) OR (antenatal) OR (ante natal) OR (intrapartum) OR (intra partum) OR (birth) OR (childbirth) OR (obstetric) OR (postpartum) OR (post partum) OR (perinatal) OR (peri natal) OR (postnatal) OR (post natal) IN NHSEED

#3 (health) OR (care) OR (service$) OR (facility$) OR (visit$) OR (interaction$) OR (contact$) OR (communicat$) OR (relation$) IN NHSEED

#4 #2 AND #3

#5 #1 OR #4

#6 (Africa) OR (Asia) OR (Caribbean) OR (West Indies) OR (South America) OR (Latin America) OR (Central America) OR (Middle East) IN NHSEED

#7 (Afghanistan) OR (Albania) OR (Armenia) OR (Armenian) OR (Bangladesh) OR (Benin) OR (Belize) OR (Bhutan) OR (Bolivia) OR (Burkina Faso) OR (Burkina Fasso) OR (Upper Volta) OR (Burundi) OR (Urundi) OR (Cambodia) OR (Khmer Republic) OR (Kampuchea) OR (Cameroon) OR (Cameroons) OR (Cameron) OR (Camerons) OR (Cameroun) OR (Cape Verde) OR (Central African Republic) OR (Chad) OR (Comoros) OR (Comoro Islands) OR (Mayotte) OR (Congo) OR (Zaire) OR (Cote d'Ivoire) OR (Ivory Coast) OR (Djibouti) OR (French Somaliland) OR (East Timor) OR (East Timur) OR (Timor Leste) OR (Egypt) OR (United Arab Republic) OR (El Salvador) OR (Eritrea) OR (Ethiopia) OR (Fiji) OR (Gambia) OR (Gaza) OR (Georgia) OR (Georgian) OR (Ghana) OR (Gold Coast) OR (Guatemala) OR (Guinea) OR (Guam) OR (Guiana) OR (Guyana) OR (Haiti) OR (Honduras) OR (India) OR (Indonesia) OR (Iraq) OR (Kenya) OR (Kiribati) OR (Korea) OR (Kosovo) OR (Kyrgyzstan) OR (Kirghizia) OR (Kyrgyz Republic) OR (Kirghiz) OR (Kirgizstan) OR (Lao PDR) OR (Laos) OR (Lesotho) OR (Basutoland) OR (Liberia) IN NHSEED

#8 (Madagascar) OR (Malagasy Republic) OR (Malawi) OR (Nyasaland) OR (Mali) OR (Marshall Islands) OR (Mauritania) OR (Agalega Islands) OR (Micronesia) OR (Moldova) OR (Moldovia) OR (Moldovian) OR (Morocco) OR (Ifni) OR (Mozambique) OR (Myanmar) OR (Myanma) OR (Burma) OR (Nepal) OR (Netherlands Antilles) OR (New Caledonia) OR (Nicaragua) OR (Niger) OR (Nigeria) OR (Pakistan) OR (Palestine) OR (Paraguay) OR (Philippines) OR (Philipines) OR (Phillipines) OR (Phillippines) OR (Rwanda) OR (Ruanda) OR (Samoa) OR (Samoan Islands) OR (Navigator Island) OR (Navigator Islands) OR (Sao Tome) OR (Senegal) OR (Sierra Leone) OR (Sri Lanka) OR (Ceylon) OR (Solomon Islands) OR (Somalia) OR (Sudan) OR (Swaziland) OR (Syria) OR (Tajikistan) OR (Tadzhikistan) OR (Tadjikistan) OR (Tadzhik) OR (Tanzania) OR (Togo) OR (Togolese Republic) OR (Tonga) OR (Trinidad) OR (Tobago) OR (Tunisia) OR (Turkey) OR (Turkmenistan) OR (Turkmen) OR (Uganda) OR (Ukraine) OR (Uzbekistan) OR (Uzbek) OR (Vanuatu) OR (New Hebrides) OR (Vietnam) OR (Viet Nam) OR (West Bank) OR (Yemen) OR (Zambia) OR (Zimbabwe) OR (Rhodesia) IN NHSEED

#9 (developing) OR (less developed) OR (under developed) OR (underdeveloped) OR (middle income) OR (low income) IN NHSEED

#10 (countr$) OR (nation$) OR (population$) OR (world) OR (econom$) IN NHSEED

#11 #9 AND #10

#12 (low$) IN NHSEED

#13 (gdp) OR (gnp) OR (gross domestic) OR (gross national) IN NHSEED

#14 #12 AND #13

#15 #6 OR #7 OR #8 OR #11 OR #14

#16 #5 AND #15

POPLINE

Keyword search: Cost effectiveness.kw AND Maternal-Child Health Services.kw

Global search: (cost*) AND (maternal or maternity or neonatal or newborn) AND (care or service*)

Selected Organizations

The websites of the following organizations were manually searched for eligible literature:

- Alliance for Health Policy and Systems Research
- Bernard van Leer Foundation
- Bill & Melinda Gates foundation
- Countdown to 2015 Initiative
- Global Library of Women's Medicine
- International Initiative on Maternal Morality and Human Rights
- International Planned Parenthood Federation
- John Snow International
- March of Dimes
- Maternal Health Task Force
- Healthy Newborn Network
- Partnership for Mother and Newborn Health
- Path
- Pathfinder International
- Population Council
- Save the Children
- The White Ribbon Alliance
- The Women's Global Network for Reproductive Rights
- UNICEF
- WHO
- Women Deliver
