# Supplementary material for: Cost-effectiveness of strategies to improve the utilization and provision of maternal and newborn health care in low-income and lower-middle-income countries: a systematic review
Source: BMC Pregnancy Childbirth. 2014 Jul 22;14:243. doi: 10.1186/1471-2393-14-243 (PMC4223592; doi:10.1186/1471-2393-14-243)
Supplement: Additional file 2 — List of eligible countries. [file 1471-2393-14-243-S2.pdf]

Additional File B: List of eligible countries

To be included in the review the study must have taken place in one or more low-income or lower-middle income countries, as classified by the World Bank in 2012. (http://data.worldbank.org/about/country-classifications/country-and-lending-groups)

The following countries were low-income countries (with GDP per capita of $1025 or less) in 2012:

- Afghanistan;
- Bangladesh;
- Benin;
- Burkina Faso;
- Burundi;
- Cambodia;
- Central African Republic;
- Chad;
- Comoros;
- Democratic Republic of Congo;
- Eritrea;
- Ethiopia;
- The Gambia;
- Guinea;
- Guinea-Bissau;
- Haiti;
- Kenya;
- Democratic Republic of Korea;
- Kyrgyz Republic;
- Liberia;
- Madagascar;
- Malawi;
- Mali;
- Mauritania;
- Mozambique;
- Myanmar;
- Nepal;
- Niger;
- Rwanda;
- Sierra Leone;
- Somalia;
- Tajikistan;
- Tanzania;
- Togo;
- Uganda; and
- Zimbabwe.

The following countries were lower-middle-income countries (with GDP per capita of $1026 to $4035) in 2012:

- Albania;
- Armenia;
- Belize;
- Bhutan;
- Bolivia;
- Cameroon;
- Cape Verde;
- Republic of Congo;
- Cote d'Ivoire;
- Djibouti;
- Egypt;
- El Salvador;
- Fiji;
- Georgia;
- Ghana;
- Guatemala;
- Guyana;
- Honduras;
- Indonesia;
- India;
- Iraq;
- Kiribati;
- Kosovo;
- Lao PDR;
- Lesotho;
- Marshall Islands;
- Micronesia, Fed. Sts.;
- Moldova;
- Morocco;
- Nicaragua;
- Nigeria;
- Pakistan;
- Papua New Guinea;
- Paraguay;
- Philippines;
- Samoa;
- Sao Tome and Principe;
- Senegal;
- Solomon Islands;
- South Sudan;
- Sri Lanka;
- Sudan;
- Swaziland;
- Syrian Arab Republic or Syria;
- Timor-Leste;
- Tonga;
- Ukraine;
- Uzbekistan;
- Vanuatu;
- Vietnam;
- West Bank and Gaza;
- Yemen; and
- Zambia
